# Nitric Oxide Synthase (NOS) Inhibition during Porcine *In Vitro* Maturation Modifies Oocyte Protein S-Nitrosylation and *In Vitro* Fertilization

**DOI:** 10.1371/journal.pone.0115044

**Published:** 2014-12-26

**Authors:** Jon Romero-Aguirregomezcorta, Ángela Patricia Santa, Francisco Alberto García-Vázquez, Pilar Coy, Carmen Matás

**Affiliations:** 1 Department of Physiology, Veterinary Faculty, University of Murcia, International Excellence Campus for Higher Education and Research (Campus Mare Nostrum), Murcia, Spain; 2 Institute for Biomedical Research of Murcia (IMIB), Murcia, Spain; Institute of Zoology, Chinese Academy of Sciences, China

## Abstract

Nitric oxide (NO) is a molecule involved in many reproductive processes. Its importance during oocyte *in vitro* maturation (IVM) has been demonstrated in various species although sometimes with contradictory results. The objective of this study was to determine the effect of NO during IVM of cumulus oocyte complexes and its subsequent impact on gamete interaction in porcine species. For this purpose, IVM media were supplemented with three NOS inhibitors: NG-nitro-L-arginine methyl ester (L-NAME), NG-monomethyl-L-arginine (L-NMMA) and aminoguanidine (AG). A NO donor, S-nitrosoglutathione (GSNO), was also used. The effects on the cumulus cell expansion, meiotic resumption, zona pellucida digestion time (ZPdt) and, finally, on *in vitro* fertilization (IVF) parameters were evaluated. The oocyte S-nitrosoproteins were also studied by *in situ* nitrosylation. The results showed that after 42 h of IVM, AG, L-NAME and L-NMMA had an inhibitory effect on cumulus cell expansion. Meiotic resumption was suppressed only when AG was added, with 78.7% of the oocytes arrested at the germinal vesicle state (P<0.05). Supplementation of the IVM medium with NOS inhibitors or NO donor did not enhance the efficiency of IVF, but revealed the importance of NO in maturation and subsequent fertilization. Furthermore, protein S-nitrosylation is reported for the first time as a pathway through which NO exerts its effect on porcine IVM; therefore, it would be important to determine which proteins are nitrosylated in the oocyte and their functions, in order to throw light on the mechanism of action of NO in oocyte maturation and subsequent fertilization.

## Introduction

One of the problems that affect in vitro fertilization (IVF) in mammals is polyspermy [1]. In porcine this problem is especially important [2] and, as a consequence, the in vitro production of embryos is extremely low, with respect to other species. For this reason most work to date has focused on studying the conditions affecting IVF [3]. However, oocyte in vitro maturation (IVM) is another important step that could be related to polyspermic fertilization and low embryo production. In this sense, it has been shown that fewer in vitro matured oocytes develop into blastocyst stage than their in vivo matured counterparts [4].

A key role in regulating oocyte maturation has been demonstrated for nitric oxide (NO) [5], an important component of the oocyte microenvironment, which effectively functions to delay oocyte aging (aged oocytes promote polyspermy [6]). The variable NO levels measured inside oocytes [7], could also affect IVM and IVF. Moreover, NO has been suggested to act as an intracellular signal that triggers the activation of the oocyte [8].

In contrast to many other molecules whose signaling mechanisms and biological effects have been studied for many years, the NO-signaling processes have only recently begun to be studied. Despite its molecular simplicity, NO acts as a biological signal in a number of ways [9]. NO, a gas that acts as a messenger molecule, is very unstable and short-lived, and it diffuses to any point of the cell membrane. It is generated from molecular oxygen and L-arginine by nitric oxide synthase (NOS), forming citrulline and NADP^+^ [10]–[12]. There are three NOS isoforms, which can be found in a variety of cell types, and more than one isoform can be expressed by a given cell type [13]. Neuronal NOS (nNOS or NOS type I) and endothelial NOS (eNOS or type III NOS), also referred to as constitutive NOS, are responsible for the continuous basal release of NO. These isoforms are independent of the physiologic demand and require calcium/calmodulin activation [11], [14]. A third isoform, inducible NOS (iNOS or NOS type II), which is calcium-independent, is expressed in response to inflammatory cytokines and lipopolysaccharide [15]. All three NOS isoforms have been identified in the ovary [16] and are involved in ovarian follicular development [17], oocyte meiotic maturation [18]–[21], oocyte activation, fertilization and embryo implantation in the uterus [8], [11].

Nitric oxide plays a dual role in reproduction, depending on its concentration. At low concentrations it stimulates or enhances early reproductive events, but both an excess and a lack of NO have negative consequences [22], [23]. In mammalian oocytes, under in vitro conditions, it has been found that high concentrations of NO inhibit meiotic maturation, produce oxidative stress and apoptosis [17], [22], [24], while low concentrations protect against oxidative stress, stimulate meiotic maturation [8], [16], [25], [26] and extend the “oocyte temporal window for optimal fertilization and development” [27].

The literature contains several studies on the effect of NO on oocyte maturation. However, in porcine species, such studies are very limited and do not take into account the repercussions on IVF parameters. IVM in pig is a long process, during which free radicals are generated [28]. For this reason, our starting hypothesis was that if NO synthesis during IVM could be decreased, better maturation and, consequently, improved IVF parameters would be achieved. To validateor rejectourhypothesis we performed IVM with three NOS inhibitors: aminoguanidine (AG) (which selectively inhibits iNOS [29]), NG-nitro-L-arginine methyl ester (L-NAME) (which preferentially inhibits eNOS [30], [31]) and NG-monomethyl-L-arginine (L-NMMA) (which inhibits NO synthesis from the three isoforms [11]); we also used an endogenous NO donor, S-nitrosoglutathione stable analog (GSNO). The effect on cumulus cell (CC) expansion, meiotic resumption, zona pellucida (ZP) digestion time (ZPdt) and finally, IVF parameters [sperm binding to the ZP (SPZ-ZP), sperm penetration (PEN), number of sperm per oocyte (SPZ-OO) and monospermy rate (MON)] were evaluated.

Furthermore, NO has the potential to induce protein S-nitrosylation [32], a reversible post-translational modification that involves the attachment of a NO moiety to a protein sulfhydryl group. To elucidate whether the effect of NOS inhibition on IVM followed the S-nitrosylated protein pathway, we studied oocyte *in situ* S-nitrosylation under different experimental conditions.

## Materials and Methods

This study was developed following institutional approval from the Bioethics Committee of the University of Murcia, and was performed in accordance with the Animal Welfare regulations of that institution.

### Materials

Unless otherwise stated, chemicals and reagents were purchased from Sigma-Aldrich Química S.A. (Madrid, Spain). Equine chorionic gonadotropin (eCG; Foligon) was supplied by Intervet International BV (Boxmeer, Holland), human chorionic gonadotropin (hCG; VeterinCorion) by Divasa Farmavic (Barcelona, Spain) and Percoll by GE Healthcare (Uppsala, Sweden). Texas Red-2-sulphonamidoethyl methanethiosulphonate (MTSEA-Texas Red) was obtained from Toronto Research Chemicals (North York, Ontario, Canada) and the prolonged antifade mounting medium (SlowFadeAntifade Kit) by Invitrogen (Paisley, United Kingdom). N^G^-nitro-L-arginine methyl ester (L-NAME; 483125) and N^G^-monomethyl-L-arginine (L-NMMA; 475886) were purchased from Calbiocherm (distributed by Merck Chemicals, Beeston, Notthingan, UK)

### Culture media

The medium used for the IVM of pig oocytes was NCSU-37 [33] supplemented with 0.57 mM cysteine, 1 mM dibutyryl cAMP, 5 mg/ml insulin, 50 µM β-mercaptoethanol, 10 IU/ml eCG, 10 IU/ml hCG and 10% porcine follicular fluid (v/v).

The basic medium used for pig IVF was TALP medium [34], consisting of 114.06 mM NaCl, 3.2 mM KCl, 8 mM Ca-Lactate.5H_2_O, 0.5 mM MgCl_2_.6H_2_O, 0.35 mM NaH_2_PO_4_, 25.07 mM NaHCO_3_, 10 mM Na lactate, 1.1 mM Na pyruvate, 5 mM glucose, 2 mM caffeine, 3 mg/ml BSA (A-9647), 1 mg/ml polyvinyl alcohol (PVA), and 0.17 mM kanamycin sulfate.

### Oocyte collection and IVM

Ovaries from Landrace x Large White gilts were collected at a local slaughterhouse (El Pozo Alimentación S.A., Alhama de Murcia, Murcia, Spain) and transported to the laboratory in saline solution containing 100 µg/ml kanamycin sulfate at 38.5°C, washed once in 0.04% cetrimide solution and twice in saline within 30 min of slaughter. Cumulus-oocyte complexes (COCs) were collected from antral follicles (3–6 mm diameter), washed twice with Dulbecco's PBS (DPBS) supplemented with 1 mg/ml PVA and 0.005 mg/ml red phenol, and twice more in maturation medium previously equilibrated for a minimum of 3 h at 38.5°C under 5% CO_2_ in air. Only COCs with complete and dense cumulus oophorus were used for the experiments. Groups of 50 COCs were cultured in 500 µl maturation medium for 22 h at 38.5°C under 5% CO_2_ in air. After culture, oocytes were washed twice in fresh maturation medium without dibutyryl cAMP, eCG, and hCG and cultured for an additional period of 20–22 h. The media were supplemented with NOS inhibitors, NO donor or not supplemented, as described below in experimental design.

### Assessment of ZP solubility

**Figure 1:**
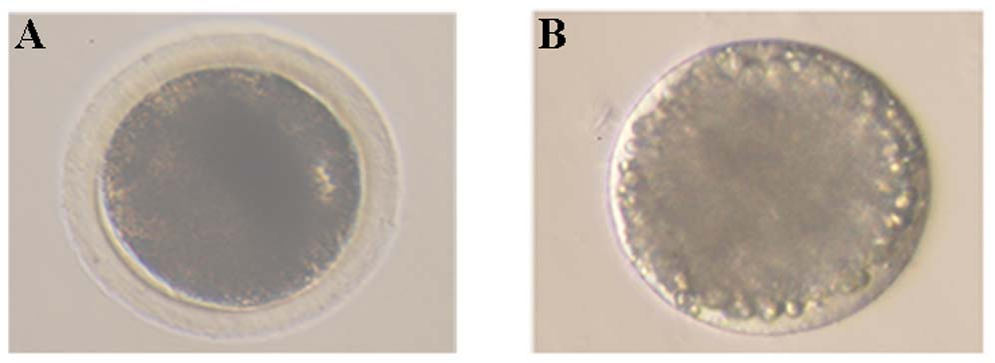
Effects of NO on ZPdt. In vitro matured porcine oocytes, after removing the CCs, were washed and transferred into drops of 0.5% (w/v) pronase solution in DPBS. ZPs were continuously observed for dissolution under an inverted microscope at 38.5°C. Representative pictures of porcine oocytes before (A) and after (B) pronase digestion.

After removing the CCs, the in vitro matured porcine oocytes with different treatments were washed quickly in DPBS and transferred into drops of 50 µl of 0.5% (w/v) pronase solution in DPBS. ZPs were continuously observed for dissolution under an inverted microscope equipped with a warm plate at 38.5°C (Fig. 1).

### IVF

COCs cultured for a total of 44 h in maturation medium from each treatment were stripped of CCs by pipetting and washed three times with TALP medium; groups of 50 oocytes were transferred into each well of a 4-well multidish containing 250 µl IVF medium previously equilibrated at 38.5°C under 5% CO_2_. The sperm-rich fraction of semen from mature, fertility-tested boars was collected by the gloved hand method, immediately transported to the laboratory and diluted at 1∶8 in Beltsville thawing solution [35]. Aliquots (0.5 ml) of semen samples from two different boars were mixed and centrifuged (740 *g*, 30 min) through a discontinuous Percoll gradient (45 and 90% v/v) and the resultant sperm pellets were diluted in TALP medium and centrifuged again for 10 min at 740 *g*. Finally, the pellet was diluted in TALP and 250 µl of this suspension were added to the wells containing the oocytes, giving a final concentration of 2.5×10^5^ cells/ml. At 18–20 h post-insemination (hpi), putative zygotes were fixed and stained for later evaluation.

### Hoechst staining

Oocytes after IVM and putative zygotes were fixed for 15 min (0.5% glutaraldehyde in DPBS), stained for 30 min (1% Hoechst 33342 in DPBS), washed in DPBS containing 1 mg/ml polyvinylpyrrolidone and mounted on glass slides. Oocytes were examined under an epifluorescence microscope at 200X and 400X magnifications.

### *In situ* protein S-nitrosylation

To visualize the S-nitrosylation of oocyte proteins a method adapted from Lefièvre et al. [36], [37] was used. Oocytes from the different experimental groups were fixed in 4% formaldehyde in DPBS (v/v) at room temperature for 30 min, washed twice with 0.1% Triton X-100 in DPBS (v/v) for 5 min to permeabilise the cells and twice with HEN (250 mM HEPES, pH 7.7, 1 mM EDTA, 0.1 mM neocuproine). Thiol groups were then blocked with 20 mM methyl methane thiosulphonate (MMTS; a thiol-reactive agent) in HEN at 50°C for 30 min. The oocytes were then washed four times with HEN, after which they were incubated with 1 mM ascorbate (to reduce the S-nitrosothiols) and 0.4 mM MTSEA-Texas Red (a fluorescent derivative of MTSEA) in HEN at room temperature for 1 h. Excess dye was removed by washing the oocytes repeatedly with 0.1% Triton X-100 in HEN. Stained oocytes were then mounted on glass slides with prolonged antifade mounting medium.

### Fluorescence intensity measurement

All images were taken using the AxioVision imaging system (Rel. 4.8) with an AxioCamHRc camera (Carl Zeiss, Göttingen, Germany) attached to a Leica DMR fluorescence microscope (Leica Microsystems, Wetzlar, Germany) equipped with fluorescent optics (N2.1 filter; excitation BP 515–560 nm). Fluorescence was measured in each oocyte using the Leica QWin image analysis software (Leica Microsystems, Barcelona, Spain) in a blind analysis.

### Statistical analysis

The data are presented as the mean ± standard error of the mean (SEM) and were tested for normality using the Kolmogorov-Smirnov test, and the homogeneity of variance was determined using the Levene test. ANOVA was used for the statistical analysis of COC diameter, ZPdt and IVF parameters and the means were separated using the Tukey test at P<0.05. To assess protein S-nitrosylation, the intensity of fluorescence in each oocyte was measured and transformed into grey values. Since the data did not satisfy the Kolmogorov-Smirnov and Levene tests, the Kruskal-Wallis test was applied, and treatment average ranks were separated using the stepwise step-down multiple comparisons method [38] at P<0.05. The true means of the data, rather than ranked means, are presented. All statistical analyses were conducted using IBM SPSS Statistics for Windows, Version 19.0 (IBM, Armonk, NY, USA).

### Experimental design

To evaluate the effect of the NO synthesis inhibition on porcine oocyte IVM and the subsequent impact on gamete interaction, five experimental groups were used: 1) CONTROL: oocytes matured without treatment; 2) GSNO: oocytes matured with 100 µM GSNO; 3) L-NAME: oocytes matured with 10 mM L-NAME; 4) L-NMMA: oocytes matured with 10mM L-NMMA and 5) AG: oocytes matured with 10 mM AG. These concentrations were chosen based on a literature review. Morphologic criteria were followed to determine whether the given NOS inhibitors or NO donor concentration caused oocyte degeneration. Oocytes with a cytoplasm of irregular appearance and dark areas were classified as degenerated [39] and removed from the experiments.

#### Effects of NO on the IVM parameters: CC expansion, meiotic resumption and ZPdt

The three NOS inhibitors and the NO donor were used to assess the effect of NO on COC maturation. To this end, CC expansion, oocyte nuclear maturation and ZP digestion were evaluated.

To evaluate the expansion of CCs, images of COC groups were takenusing a Nikon SMZ-10A stereomicroscope at 10X magnification and COC diameter was measured in each oocyte using the ImageJ software (http://imagej.nih.gov/ij) in a blind analysis. This experiment was repeated 3 times with a total of 871 COCs.

To assess nuclear maturation, oocytes from the different experimental groups were denuded, fixed and stained. When the nucleus was in germinal vesicle or metaphase I, the oocyte was considered as immature, while it was considered as mature when the metaphasic plate and polar body were present. This experiment was repeated 5 times with a total of 709 oocytes.

The dissolution time of the ZP of each oocyte was registered as the time between the placement of the oocytes in the pronase solution and the time when the ZP was no longer visible at 200X magnification. This time was referred to as ZPdt. This experiment was repeated 5 times with a total number of 605 oocytes.

#### Effect of NO during oocyte maturation on the gamete interaction

The IVF was used as a tool to determine the role of NO during oocyte IVM. For this purpose, 20–25 denuded oocytes for each experimental group were used in each trial. Maturation (%), PEN (percentage of mature oocytes with decondensed sperm heads or male pronuclei in the oolemma), SPZ-ZP, SPZ-OO and MON were assessed in each oocyte. This experiment was repeated 5 times to evaluate a total of 609 oocytes.

#### Effects of NO on S-nitrosylated proteins in oocytes

To elucidate whether the effect of NO on IVM was through the S-nitrosylated protein pathway, the *in situ* S-nitrosylation was used. The fluorescence intensity in oocytes from the same five experimental groups was compared, and all fluorescent images were digitally converted into a grey scale image before commencing the analysis [40]. To quantify the fluorescence intensity we determined the mean grey value in each oocyte, ranging from black to white (0–255). For this experiment 288 oocytes in 4 replicates were evaluated.

Oocytes processed without MMTS (all thiol groups available) were taken as positive control, while, for negative controls oocytes processed without ascorbate (absence of thiol groups) or without MTSEA-Texas Red (no fluorescent labeling) were used.

## Results

### Effect of NO on the IVM parameters: CC expansion, meiotic resumption and ZPdt

**Figure 2:**
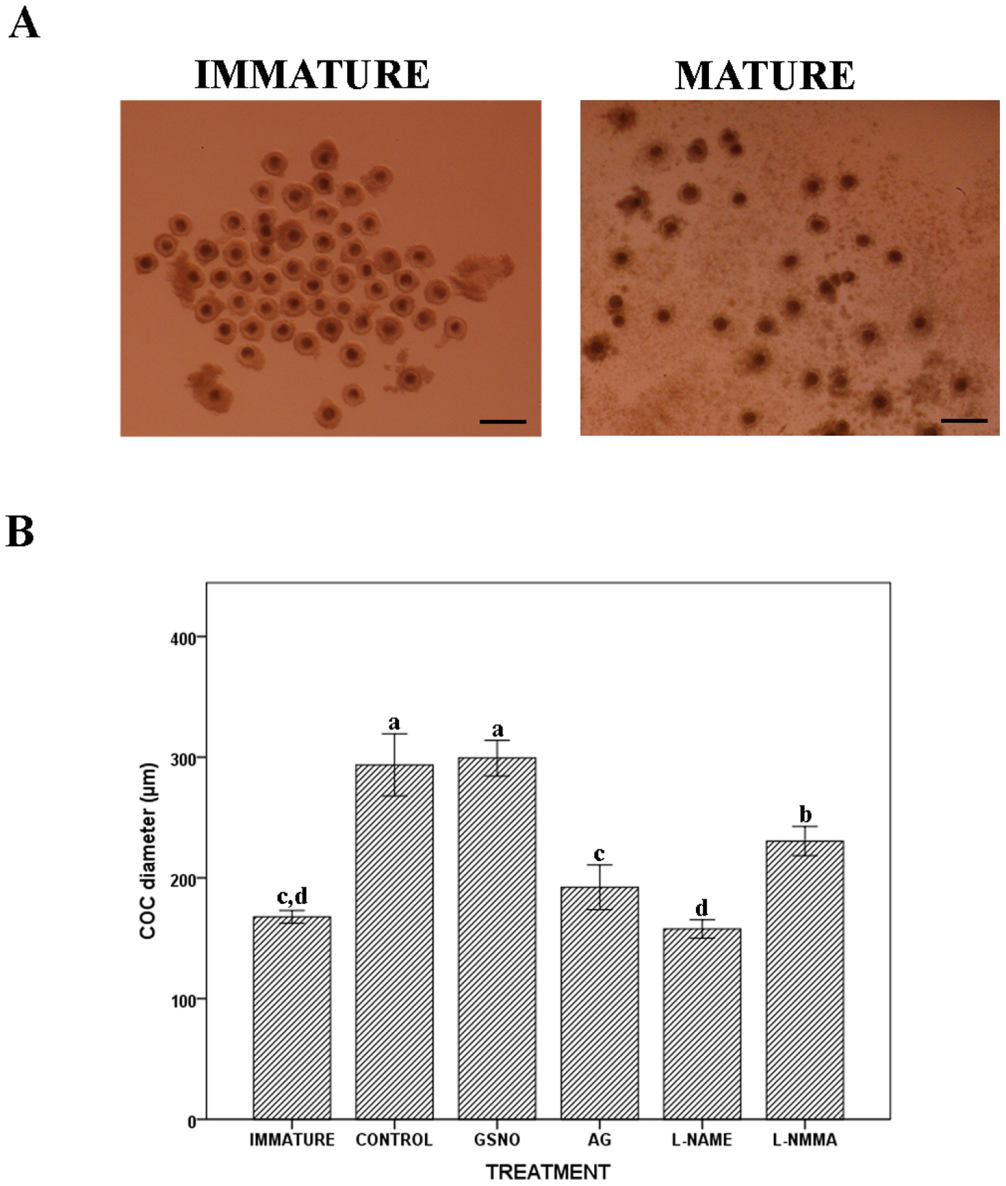
Effects of NO on the CC expansion. (A) Immature (left) and mature (right) porcine COCs. Images were taken with a Nikon SMZ-10A stereomicroscope (Magnification10X). Scale bar  = 250 µm. (B) The average diameter (mean ± SEM in µm, n of COCs) of immature porcine COCs (167.72±2.63, n = 150) was compared to COCs after 44 h in maturation medium under different experimental conditions: CONTROL (293.60±12.87, n = 141), GSNO (299.22±7.39, n = 147), AG (192.17±9.25, n = 149), L-NAME (157.66±3.79, n = 140) and L-NMMA (230.43±6.03, n = 144). The letters a,b,c in different bars denote significant differences (p<0.05).

The addition of the NO donor during IVM did not affect cumulus expansion since COC diameters in the GSNO-treated oocytes did not differ significantly from those of the control group (299.22±7.39 µm in GSNO group vs. 293.60±12.87 µm in control group, Fig. 2).

In contrast, the addition of any of the inhibitors resulted in a significantly lower degree of CCs expansion compared with the control group (192.17±9.25 µm, 157.66±3.79 µm and 230.43±6.03 µm, for AG,L-NAME and L-NMMA inhibitors, respectively P<0.05, Fig. 2). Comparisons among the three inhibitors showed that L-NAME was the most effective at reducing the COC diameter, followed by AG and LNMMA (p<0.05). When the COC diameters from the AG and L-NAME groups were compared to those measured in immature COCs before starting the maturation process (167.72±2.63 µm), no significant difference was found, meaning that AG and L-NAME completely inhibited cumulus expansion. However, the inhibition produced by L-NMMA was only partial because the level of COC expansion in this group was significantly higher than that assessed in the immature oocytes (p<0.05, Fig. 2).

**Figure 3:**
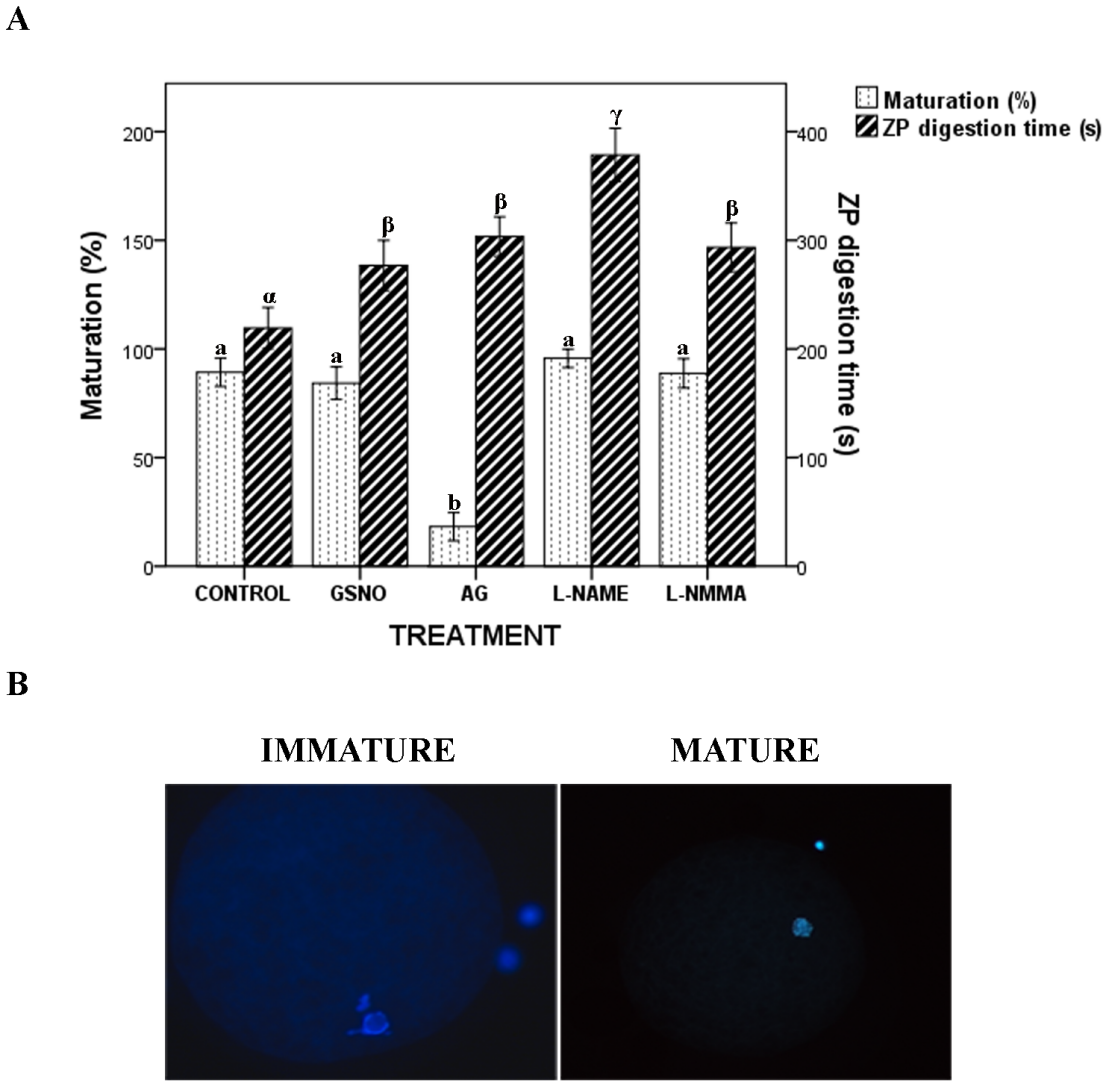
Effects of NO on the meiotic resumption. (A) Histogram showing maturation percentage (dots) and ZPdt (lines) in porcine oocytes after IVM under different experimental conditions. Letters a,b and α,β,γ in different bars denote significant differences (p<0.05). (B) Porcine oocytes were fixed, Hoechst 33342-stained, mounted on glass slides and examined under an epifluorescence microscope at 200X and 400X magnifications. Representative pictures of porcine immature (left) and matured (right) oocytes.

Meiotic resumption was not affected by the treatment with GSNO or the inhibitors L-NAME and L-NMMA (Fig. 3), with more than 87.8% of the oocytes reaching metaphase II stage. However, the AG inhibitor produced a significant reduction in this percentage, with only 17.2% of the oocytes reaching the metaphase II stage (Fig. 3) and 82.7% of the oocytes still showing the nucleus in the germinal vesicle stage (Fig. 3). The different treatments did not cause significant oocyte degeneration, which never exceeded 5% (S1 Table).

When ZP solubility was assessed a significant increase in ZPdt was observed for all the experimental groups (277.6±9.5 s, 371.8±11.3 s, 304.3±8.1 sand 295.6±10.1 sfor GSNO, L-NAME, AG and L-NMMA, respectively) compared to the control (235.5±8.8 s) (Fig. 3).

### Effect of NOS inhibition during oocyte maturation on the gamete interaction

The only NOS inhibitor significantly affecting the percentage of oocytes penetrated after IVF was L-NAME (Table 1), which reduced this percentage by about 18%. Significant, too, was the effect of the NO donor, GSNO, and the L-NAME inhibitor on the mean number of spermatozoa that remained attached to the ZP at the end of the sperm-oocyte coincubation period (p<0.05, Table 1). In both cases, fewer spermatozoa than in the control group were found attached to the ZP. By contrast, AG, the only inhibitor affecting oocyte maturation, showed a higher mean number of spermatozoa attached to ZP than the control oocytes (p<0.05, Table 1). The percentage of monospermy was not affected by any treatment. As regards cytoplasmic maturation, assessed as the percentage of oocytes able to form male pronuclei, the oocytes were able to promote pronuclear formation in all cases.

**Table 1:** Effect of NO during oocyte maturation on the later gamete interaction.

Group	N	Maturation (%)	Penetration (%)	Sperm/OO (n)	Sperm/ZP (n)	Monospermy (%)
CONTROL	119	96.64±1.66^a^	73.04±4.16^a^	3.11±0.27	25.02±1.83^a^	27.38±4.90
GSNO	106	94.34±2.26^a^	63.00±4.85^a,b^	2.67±0.27	14.36±1.07^b^	36.51±6.11
AG	132	18.94±3.42^b^	52.00±10.20^a,b^	3.31±0.80	35.28±5.72^c^	46.15±14.39
L-NAME	123	91.06±2.58^a^	45.54±4.73^b^	2.14±0.19	12.76±1.45^b^	33.33±6.67
L-NMMA	129	89.92±2.66^a^	61.21±4.54^a,b^	2.72±0.32	18.79±2.12^a,b^	49.30±5.98

a, b, c in the same column denote significant differences (P<0.05).

### Effects of NO on S-nitrosylated proteins in oocytes

**Figure 4:**
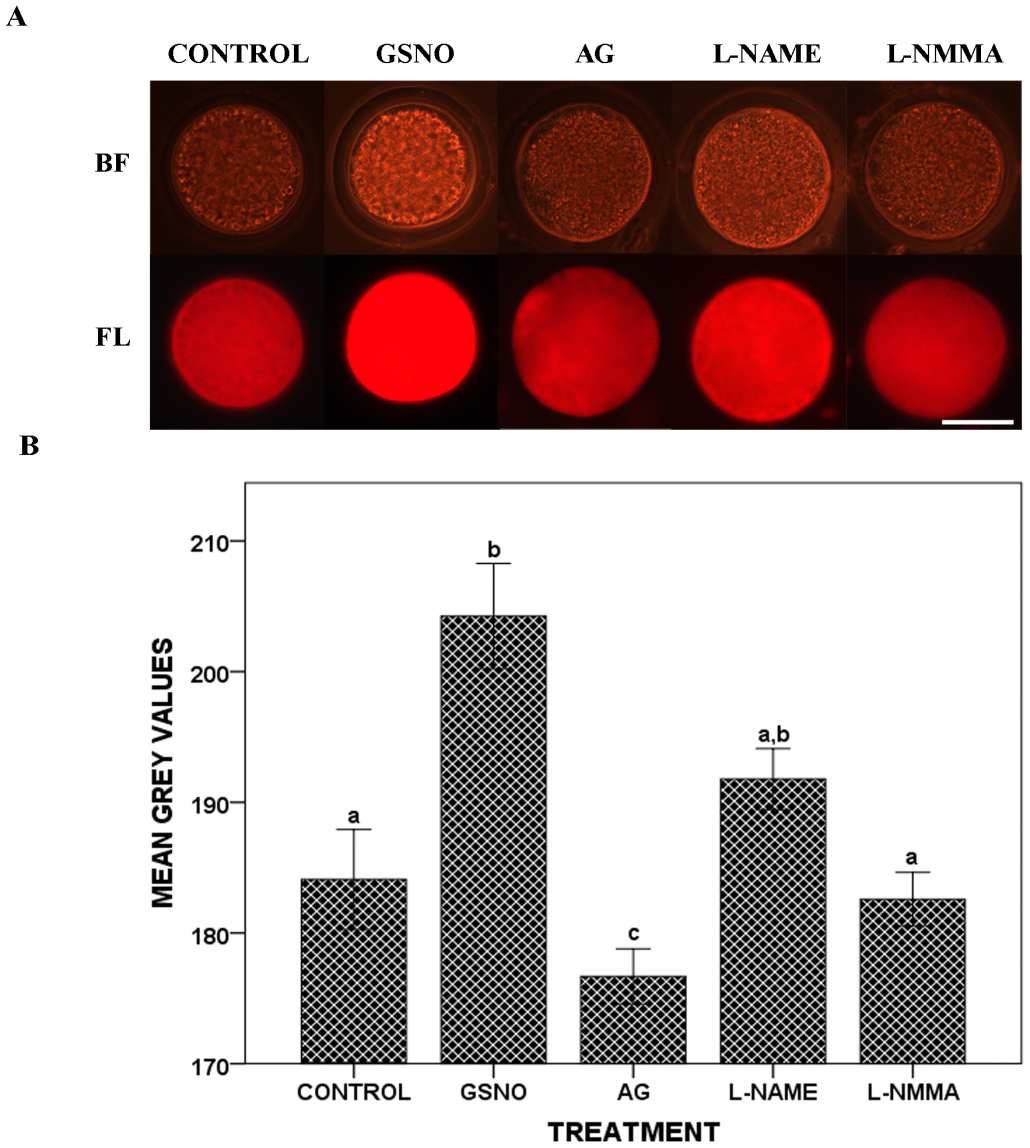
Effects of NO on S-nitrosylated proteins in oocytes. Oocytes from the different experimental groups were submitted to *in situ* protein s-nitrosylation. (A) Porcine oocytes after *in situ* S-nitrosylation. For each group the brightfield image (BF) and the fluorescent counterparts (FL) are shown. Scale bar  = 50 µm. (B) Histogram showing mean grey values after *in situ* S-nitrosylation of porcine oocytes. All fluorescent images were digitally converted into a grey scale image before commencing the analysis. To quantify the fluorescence intensity the mean grey value (0–255) was determined in each oocyte. The data are presented as the true means ± SEM rather than ranked means. The letters a,b,c in different bars denote significant differences (p<0.05).

A significant increase in the intensity of MTSEA-Texas Red labeling was visually observed in oocytes incubated in the presence of GSNO (Fig. 4). When this difference was quantified as intensity of fluorescence in arbitrary units, which in theory corresponds to the amount of oocyte S-nitrosoproteins, it was confirmed that the values for the GSNO group (204.25±4.03) were significantly higher than those for Control, L-NAME or L-NMMA groups (184.10±3.81, 191.77±2.34 and 182.59±2.06, respectively, p<0.05, Fig. 4), whereas oocytes incubated in the presence of AG inhibitor showed the lowest amount of S-nitrosoproteins (176.68±2.11, p<0.05, Fig. 4).

## Discussion

This study was conducted to ascertain whether the inhibition of NO production during maturation could improve IVF parameters. Many authors have written about the role of NO in oocyte maturation and there is much controversy concerning its function. It has been shown how NO is related to cortical granules, meiotic spindles, aging or degeneration, all factors demonstrated to affect the porcine IVF outcome [3] and, more recently, the importance of NO as a cellular messenger with effect on reproductive parameters has been demonstrated [9]. However we found no reference in the literature to the direct involvement of NO in gamete interaction or fertilization in the porcine species.

During oocyte maturation, hyaluronan is synthesized and accumulated by the CCs, remaining embeddedin a gelatinous matrix [41] and causing cumulus expansion. In our study, it was first shown that CC expansion was suppressed by iNOS and eNOS inhibitors, suggesting that NO derived from these isoforms might be necessary for the expansion of CCs. Similar results were obtained in cattle [42], [43], mice [26], sheep [44], rat [45] and pig [25], [46]. In contrast, supplementation with NO did not exert any effect, probably because the NO requirements of COCs are fulfilled at this stage.

NO has been reported to play a dual role in oocyte meiotic maturation in mice, depending on its concentration, although the mechanism by which it influences oocyte maturation has not been fully clarified. Abbasi et al. [47] suggested that the key of the arrest at the first meiotic division is the concentration of cAMP and cGMP in the preovulatory follicle. Since NO is a well known factor that stimulates cGMP production, they concluded that high NO concentration causes arrest, whereas a decrease in NO generation results in the resumption of meiosis.

By contrast, in the present paper, the addition of AG to the maturation medium prevented cumulus expansion and also inhibited nuclear maturation. However, L-NAME and to a lesser degree, L-NMMA, only inhibited CC expansion and not nuclear or cytoplasmic maturation, as can be deduced from the later ability to form male pronuclei. Different hypotheses can be proposed to explain these results. First, it is known that during the IVM of porcine oocytes the follicle-stimulating hormone (FSH) increases both prostaglandin (PG) E2 production and the expression levels of EGF-like factors [48]. It is plausible that PGE2, which plays an indirect role in stimulating the expansion of mouse CCs, is stimulated by NO, as was demonstrated in rabbits [49], where the PGE2 and PGF2α production in the ovary in response to hCG is blocked by L-NAME. Considering these results, we propose that the effect of NOS inhibitors on CC expansion may be related to the suppression of PG production by the induced decrease in NO levels.

Second, the fact that the addition of L-NAME to maturation media did not allow cumulus expansion while the oocytes resumed meiosis contradicts previously reported data [21], [25] that suggested the resumption of meiosis was inhibited during IVM when L-NAME was used. This could be due to methodological differences, because Tao et al. [25] used the same medium for the whole maturation time and their maturation percentage in control group was below 58%, while our figure was always above 90%; moreover, they used hypoxanthine as an inhibitor of nuclear maturation during culture (48 h), while we used dibutyryl cAMP only for the first 22 h of maturation. Another possible explanation could be that L-NMMA slightly inhibited CC expansion through its action on both NOS isoforms, but that the NO threshold for oocyte maturation was reached; although L-NAME inhibited eNOS NO production, iNOS was able to supply the NO requirements for nuclear and cytoplasmic maturation. This would agree with the observation in the present study that AG was the only inhibitor that efficiently avoided the resumption of meiosis, which would be dependent on iNOS inhibition and could not be counteracted with eNOS NO production since eNOS isoforms are independent of the physiologic demand. In addition, abnormal iNOS activity can up-regulate arginase activity, allowing it to compete with eNOS for L-arginine, resulting in reduced NO bioavailability [50].

It has been shown that ovulated porcine oocytes collected from the oviduct require more time for ZP digestion (hours) than immature or in vitro matured oocytes (minutes), hence the porcine ZP resistance to enzymatic digestion that occurs in the oviduct is independent of fertilization [51]. In our experiment, although we found a significant increase in ZPdt in all treatments, this increase was lower than the values observed in the oviductal oocytes. NO contributes to the modification of proteins through protein cysteine nitrosylation (S-nitrosylation), the covalent attachment of NO to the thiol group of cysteine [32]. Since there are a number of cysteines in the ZP glycoproteins that could be affected by NO attachment, a reduction in disulfide bonds among ZP proteins and a decrease in the ZP resistance to protease digestion might be expected. In the same way, a reduction in ZPdt when NOS NO production was inhibited would be expected. However, ZPdt increased when NOS inhibitors were added. Explanations for this fact could include the premature cortical granule exocytosis and subsequent ZP hardening induced by an insufficient amount of NO [7] although additional studies would be necessary to demonstrate such a hypothesis. Whatever the case, the mechanism that leads to ZP hardening appears to be different between NOS inhibitors and NO donor.

Reactive oxygen species (ROS) and reactive nitrogen species (RNS) are physiologically generated during in vivo oocyte maturation [52], while, in vitro, these reactive species are also generated but differently regulated. This leads to an uncontrolled reactive species production that could subsequently have an effect on IVF. Our results are consistent with this hypothesis since the penetration rate and sperm ZP binding were modified when NO generation was altered during the maturation step.

The lower penetration rate found in the L-NAME group, despite the presence of the three NOS isoforms in porcine oocytes [20], could indicate that eNOS is the isoform most directly involved in gamete interaction, as previously suggested [21]. The decrease in the number of spermatozoa bound to ZP in this group would be consistent with the increase observed in ZPdt since such a correlation has been observed in other studies [51]. How L-NAME modifies ZP glycoproteins, leading to a reduction in sperm binding and penetration, remains to be elucidated.

Nitric oxide has been reported to act on NO-sensitive guanylcyclases [53], but newlines of evidence suggest that NO can also signal through non-cGMP-mediated pathways [54] by means of protein S-nitrosylation [32], [55]. This is a redox-dependent, thiol-based, reversible post-translational protein modification that involves attachment of a NO moiety to a protein sulfhydryl group. NO gas and NO donors have the potential to induce S-nitrosylation of proteins. NO can induce S-nitrosylation in lipid membrane-rich organelles such as the mitochondria and the endoplasmic reticulum [23]

Protein S-nitrosylation regulates the activity of a number of metabolic enzymes, oxidoreductases, proteases, protein kinases and phosphatases in vivo and in vitro, as well as respiratory proteins, receptor/ion channels and transporters, cytoskeletal and structural components, transcription factors, regulatory elements (including G proteins), and others [56], [57]. Focusing on reproductive events, Bilodeau-Goeseels et al. [54] suggested, for the first time, that the inhibitory effect of NO on bovine oocyte meiotic resumption did not appear to be mediated by the cGMP/PKG pathway, in contrast to previous observations in mouse. In addition, it has been recently reported that protein S-nitrosylation occurs during in vitro embryo culture in mice [23].

Despite the above reported studies, there is still a lack of data reporting S-nitrosylation in porcine oocyte maturation. For this reason it was evaluated in this study using a method for selectively labeling S-nitrosylated proteins with a fluorescent tag. It was observed that protein S-nitrosylation only decreased in oocytes matured with AG, the iNOS inhibitor, while the highest value was obtained with the NO donor GSNO. How exactly the decrease in protein nitrosylation is related to maturation is still unknown, but, what is clear from our results is that the inhibition of iNOS during maturation (by AG) produces a lower S-nitrosylation rate in porcine oocytes and that these poorly S-nitrosylated oocytes show a lower ability to resume meiosis. These results agree with Lee et al. [23], who reported that the use of a NOS inhibitor to eliminate NO production had deleterious effects on embryo development. However, we still cannot conclude that NOS inhibition in porcine IVM has detrimental effects on the developmental ability of the oocyte, until it becomes clear which proteins are nitrosylated in the oocyte and what their functions are. This identification will provide novel insight into the mechanism of action of NO in oocyte maturation and, subsequently, fertilization.

To sum up, our results show that AG inhibits iNOS NO production, decreasing the amount of S-nitrosylated proteins and reducing meiotic resumption; so, it can be concluded that iNOS activity is necessary for proper porcine oocyte maturation. We also demonstrate that NOS inhibition during IVM prevented cumulus expansion and did not improve the IVF system but did reveal the importance of NO in maturation and subsequent fertilization. Furthermore, we report for the first time that protein S-nitrosylation acts as a pathway through which NO exerts its effect on porcine oocyte IVM.

Further studies should be performed to understand the effect of NO production inhibition not only at oocyte level but also at sperm cell level. Gamete protein S-nitrosylation should also be studied in depth to determine the involvement of this posttranslational modification on gamete interaction.

## Supporting Information

S1 Table **Effect of NO on the IVM parameters: Oocyte degeneration.**
(DOCX)
