# Supplementary material for: Nitric Oxide Synthase (NOS) Inhibition during Porcine In Vitro Maturation Modifies Oocyte Protein S-Nitrosylation and In Vitro Fertilization
Source: PLoS One. 2014 Dec 26;9(12):e115044. doi: 10.1371/journal.pone.0115044 (PMC4277276; doi:10.1371/journal.pone.0115044)
Supplement: S1 Table — Effect of NO on the IVM parameters: Oocyte degeneration. (DOCX) [file pone.0115044.s001.docx]

**Table S1. Effect of NO on the IVM parameters: Oocyte degeneration.**

| Group | N | Degeneration (%) |
| --- | --- | --- |
| CONTROL | 267 | 1.87±0.83 |
| GSNO | 267 | 4.87±1.32 |
| AG | 289 | 2.42±0.91 |
| L-NAME | 269 | 2.60±0.97 |
| L-NMMA | 266 | 2.26±0.91 |

No significant differences were found (P<0.05).
